# Surviving the Heat: Genetic Diversity and Adaptation in Sudanese Butana Cattle

**DOI:** 10.3390/genes16121429

**Published:** 2025-11-30

**Authors:** Guilherme B. Neumann, Paula Korkuć, Siham A. Rahmatalla, Monika Reißmann, Elhady A. M. Omer, Salma Elzaki, Gudrun A. Brockmann

**Affiliations:** 1 Albrecht Daniel Thaer-Institute for Agricultural and Horticultural Sciences, Animal Breeding and Molecular Genetics, Humboldt-Universität zu Berlin, Unter den Linden 6, 10099 Berlin, Germany; neumann@izw-berlin.de (G.B.N.); korkuc@izw-berlin.de (P.K.); siham.rahmatalla@hu-berlin.de (S.A.R.); monika.reissmann@hu-berlin.de (M.R.); salmafaroug@hotmail.com (S.E.); 2 Leibniz Institute for Zoo and Wildlife Research, Alfred-Kowalke-Straße 17, 10315 Berlin, Germany; 3 Department of Dairy Production, Faculty of Animal Production, University of Khartoum, P.O. Box 32, Khartoum North 13314, Sudan; 4 Department of Genetics and Animal Breeding, Faculty of Animal Production, University of Khartoum, P.O. Box 32, Khartoum North 13314, Sudan; alhadibkr@gmail.com

**Keywords:** heat stress, genetic variation, disease resistance, gene pool, inbreeding

## Abstract

Background: Butana are native Sudanese *Bos indicus* cattle that are well adapted to arid environments and valued for their relatively high milk performance and resilience under harsh conditions. Despite their adaptive advantages, Butana cattle face the risk of genetic erosion due to low production performance and the absence of structured breeding programs underscoring the urgent need to conserve their unique genetic potential for climate-resilient livestock development. Methods: In this study, we analyzed whole-genome sequencing data from 40 Butana cattle to assess their genetic diversity, population structure, signatures of selection, and potential pathogen load. Results: Butana cattle exhibited high nucleotide diversity and low levels of inbreeding, indicating a stable gene pool shaped by natural selection rather than by intensive breeding. Signatures of selection and functional variant analysis revealed candidate genes involved in heat stress adaptation (*COL6A5*, *HSPA1L*, *TUBA8*, *XPOT*), metabolic processes (*G6PD*, *FAM3A*, *SLC10A3*), and immune regulation (*IKBKG*, *IRAK3*, *IL18RAP*). Enrichment analyses and RoH island mapping consistently highlighted immune and thermoregulatory pathways as key selection targets, distinguishing Butana from both the geographically neighbored Kenana cattle and the specialized dairy cattle breed Holstein. Furthermore, metagenomic screening of unmapped reads detected the tick-borne parasite *Theileria annulata* and the opportunistic pathogen *Burkholderia cenocepacia* in all animals, underscoring the importance of integrating pathogen surveillance into genomic studies. Conclusions: Taken together, our findings highlight the distinct adaptive genomic profile of Butana cattle and reinforce their value in breeding programs aimed at improving climate resilience and disease resistance in livestock through the utilization of local breeds.

## 1. Introduction

Butana cattle are a native dairy *Bos indicus* breed of Sudan, primarily distributed in central Sudan, particularly in the Butana region along the Nile River, the Atbara River, and east of the Blue Nile [1,2]. As one of the largest East African Zebu breeds, Butana are well-known for their adaptability to arid environments, resilience under nutritional stress, and greater resistance to tick infestation compared to *Bos taurus* cattle [2,3]. Their shiny coats help protect them from the intense sun and heat, while their slender bodies, sturdy hooves, and lighter bone structure enable them to thrive in desert-like regions and endure long-distance migrations across harsh scrublands [4].

Butana cattle are especially valued for their milk yield, and high fat (6.01%) and protein content (3.74%) [5], making them an important source of nutrition for local communities [2,6]. Despite their adaptive advantages and relatively high milk yield under local conditions (4.15 ± 1.40 kg/day), Butana cattle exhibit lower overall production performance compared to Butana × Holstein crossbred cattle (10.12 ± 1.10 kg/day) [5]. As a result, they are increasingly at risk due to breed substitution, indiscriminate crossbreeding, and the absence of structured breeding programs. Amid climate change and the increasing global demand for high-quality protein, conserving and understanding locally adapted breeds is crucial. These cattle contribute to food security and provide valuable insights into the genetic basis of resilience to heat, disease, and nutritional stress.

Previous genomic studies on Butana cattle have largely focused on individual candidate genes, including *DGAT1* (diacylglycerol-O-acyltransferase) [5,7,8,9], leptin [9], caseins [9,10], growth and reproductive hormone [9,11], and the bovine leukocyte antigen complex [12], as well as traits such as mastitis resistance [13]. Additionally, Butana cattle have also been shown to share a close genetic relationship with Kenana and other African indicine breeds, such as East African Shorthorn Zebu and Sheko [14,15].

Previous efforts to identify adaptive genomic signatures in Butana cattle revealed selection on pathways related to immunity, reproduction, and heat tolerance and prioritized 87 candidate genes under positive selection in Butana [14]. However, that study relied on the Illumina BovineHD BeadChip, which had been primarily developed for commercial *B. taurus* breeds and may, therefore, miss genetic variation relevant to indicine cattle [16]. A more recent study using whole-genome sequencing across Sudanese zebu populations identified selection signals in genes involved in insulin signaling and fat metabolism [17], but treated Sudanese breeds collectively, leaving the specific genomic uniqueness of Butana unaddressed.

Besides Butana, Kenana cattle also represent an important dairy breed with high potential for milk production, growth, and reproductive efficiency under harsh environments [2,3]. Although the two breeds are genetically closely related [14,15], they are raised in ecologically distinct environments: Butana cattle are predominantly managed in arid desert zones, whereas Kenana cattle are kept in comparatively more favorable and less arid regions [2,6,17]. These environmental contrasts, along with differing management practices, are expected to have driven divergent adaptive trajectories and selection pressures between the two breeds.

This study aimed to comprehensively characterize the genomic diversity and inbreeding levels within the Butana breed, assess its genetic relationship with other indicine breeds, and identify genomic regions under selection that may have contributed to its adaptation to hot, resource-limited environments. Additionally, we explored the presence of pathogen-related sequences in the genomic data to uncover potential microbial challenges affecting this breed.

## 2. Materials and Methods

### 2.1. Population and Genotypic Data

Samples from 22 unrelated Butana cows were collected across eight different locations in the North of Sudan. Genomic DNA was extracted from blood and sequenced using the Illumina NovaSeq 6000 platform (Illumina, San Diego, CA, USA), 150 bp paired-ed (PE), yielding an average coverage of 14×. Data pre-processing, sequence alignment, variant calling and variant recalibration (with 99% tranche filtering) were performed according to the 1000 Bull Genomes Project guidelines [18]. The *B. taurus* genome version ARS-UCD1.2_Btau5.0.1Y was used as reference [19,20]. Additionally, sequence variants from 18 Butana, 17 Kenana, 274 *B. indicus* cattle representing 25 breeds, and 50 Holstein cattle (as a taurine outgroup) were obtained from the 1000 Bull Genomes Project (Run 9) and merged with our Butana dataset. Only breeds with at least five individuals sequenced at ≥5× coverage were included. In total, 54,532,198 segregating variants were retained for downstream analysis.

### 2.2. Relationship Analysis

Pairwise F_ST_ values between Butana and each of the other breeds were calculated using variants segregating in either Butana or the respective comparison breed. F_ST_ values were estimated in windows of 10 kb based on Hudson’s method [21,22] using the scikit-allel v1.3.1 library in Python [23]. A maximum likelihood tree was constructed using Treemix v1.13 [24] in blocks of 1000 variants based on a pruned dataset of 19,094,509 variants. Linkage disequilibrium (LD) pruning was performed in PLINK v2.0 [25] using an *r^2^* threshold of 0.6, a window size of 50 variants, and a step-size of 5 variants. A consensus tree was generated by bootstrapping across 1000 replicates (seed = 389) using PHYLIP v3.697 [26], and visualized with the BITE v2 package [27]. The optimal number of migrations edges was determined using the Evanno method [28] as implemented in the R package OptM v0.1.6 [29], testing migration values (m) from 0 to 10 across ten replicates. The highest Δm = 2.66 was observed at m = 3 edges, which was selected for further analysis. To assess gene flow, f3-statistics were calculated with Treemix v1.13 over blocks of 10,000 variants using the pruned dataset. The f3-statistics was calculated as f3(A; B, C), where a significantly negative value of the f3 statistic implies that population A is admixed from populations B and C. Principal Component Analysis (PCA) was performed on the pruned dataset using the scikit-learn v1.0.2 library in Python.

### 2.3. Diversity and Inbreeding Analyses

Nucleotide diversity (π) was calculated using scikit-allel v1.3.1 library in Python in windows of 10 kb (π_window_). The mean nucleotide diversity per chromosome (π_ChrMean_) was calculated as the mean of all π_window_ values of the respective chromosome and the total mean nucleotide diversity (π_TotMean_) was derived from the average of all π_window_ values across all autosomes. Only pure Butana individuals were considered for diversity, inbreeding, and signatures of selection analyses; seven animals that were clustering together with Kenana and Begait cattle in the PCA were excluded. All segregating variants in Butana were annotated using the Ensembl Variant Effect Predictor (VEP) software v105.0 [30]. Additionally, variants that segregated in Butana, but were fixed in all other investigated breeds were defined as variants unique to Butana. Gene Ontology (GO) enrichment analysis for variants of candidate genes was performed using g:Profiler version e112_eg59_p19_25aa4782 [31]. Adjusted *p*-values (*p_adj_)* <0.05 were considered significant.

Inbreeding was estimated using the excess of homozygosity (F_Hom_) and the inbreeding coefficient F_RoH_ based on runs of homozygosity (RoHs). Observed and expected homozygous genotype counts were derived using vcftools v0.1.15 [32] and F_Hom_ was calculated as:

(1) $$F_{HOM}=\frac{number\;of\;observed\;homozygous-number\;of\;expected\;homozygous}{total\;number\;of\;variants-number\;of\;expected\;homozygous}$$

F_RoH_ was estimated using BCFtools v1.9 [33] with an assumed recombination rate of 10^–8^ per base pair (1 cM/Mb). RoHs were classified into five length categories with the minimal lengths of 50 kb, 100 kb, 1 Mb, 2 Mb and 4 Mb. F_RoH_ was calculated for each group as:

(2) $$F_{RoH}=\sum \frac{L_{RoH}}{L_{genome}}$$

where L_RoH_ is the length of a homozygous region and L_genome_ the length of the genome covered by variants (2,626,672,074 bp for our dataset).

### 2.4. Signatures of Selection

To detect signatures of selection within the sequenced Butana cattle, RoH islands were defined as regions with the highest variant frequency within RoHs ≥ 50 kb. The top 0.05 percentile of variant frequencies within these RoHs was used as the threshold to define RoH islands. Only pure Butana individuals were considered in this analysis. Between populations, the cross-population-extended haplotype homozygosity (XP-EHH) [34] was calculated for pure Butana versus Kenana, and pure Butana versus Holstein using the R package rehh v3.2.2 [35]. All segregating variants in pure Butana, Kenana, or Holstein were included. Missing variants were initially imputed and phased using Beagle v5.1 [36].

Positive XP-EHH scores represent variants positively selected in pure Butana compared to Kenana or Holstein, while negative scores correspond to variants positively selected in Kenana or Holstein compared to pure Butana. After multiple testing using Bonferroni correction, *p*-values < 0.05 were considered significant. XP-EHH regions were defined by merging significant neighbouring variants and subsequently used for gene annotation. RoH islands and XP-EHH regions start and end positions were scanned for protein-coding genes using the Ensembl database release 114. Gene Ontology (GO) enrichment analysis was performed using g:profiler with the same *p*-value threshold.

### 2.5. Detection of Pathogens

The strategy for detecting pathogen sequences in whole-genome sequencing data has been described previously [37]. Similarly, the unmapped reads from sequenced Butana animals were extracted with samtools v1.20 [38] and used as input in the Kraken v2.1.3 [39], filtering for results with a confidence of at least 0.7. Relative abundances were estimated using bracken v3.0.1 [40]. For taxonomic classification, unmapped reads were classified using the core_nt database which includes GenBank, RefSeq, TPA and PDB databases and retrieved and indexed in December 2024 by the Langmead Lab (https://benlangmead.github.io/aws-indexes/, accessed on 21 January 2025).

## 3. Results

### 3.1. Relationship Among Indicine Cattle Breeds

**Figure 1:**
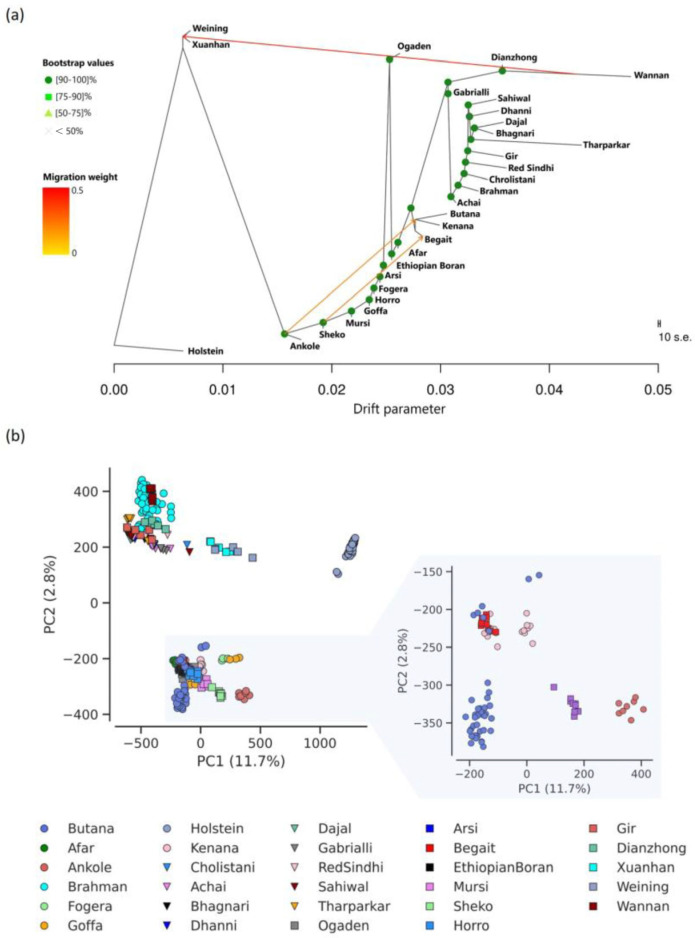
Relationship analysis among *Bos indicus* cattle breeds. (**a**) Maximum likelihood tree with Holstein as the outgroup. Green dots represent bootstrap percentages. Arrows indicate migration direction colored from yellow to red based on migration weight. The distance between the breeds is explained on the *x*-axis by drift. (**b**) Principal Component Analysis (PCA) among the breeds for the first two principal components. A detailed zoom-in is provided for the breeds Butana, Begait, Kenana, Mursi, and Ankole.

Breeds from Sudan and Ethiopia, such as Kenana, Begait, Fogera, Afar, Arsi, Horro, Ethiopian Boran, and Goffa, showed the closest genetic relationship to Butana, as supported by the maximum likelihood tree (Figure 1a), PCA (Figure 1b), and pairwise F_ST_ values (Table S1).

Pairwise F_ST_ values calculated relative to Butana ranged from 0.032 for Begait to 0.181 for Chinese Wannan and reached 0.317 for Holstein, which served as the outgroup. Furthermore, in total, three migration events were detected (Figure 1a). First, a migration from an ancestor of Wannan to a common ancestor of Weining and Xuanhan was observed. In addition, two moderate migration events were observed from an ancestor of Ankole to a common ancestor of Butana and Kenana, and from an ancestor of Sheko to Begait. However, gene flow from Ankole into Butana (f3 = 0.003, *p* = 1.20 × 10^−282^) and into Kenana (f3 = 0.002, *p* = 3.22 × 10^−94^) could not be confirmed by f3 statistics, but admixture of Butana with Holstein was observed in several cases (Table S2). The other migrations from Wannan to a common ancestor of Weining (f3 = 0.002, *p* = 3.68 × 10^−34^) and Xuanhan (f3 = −0.000, *p* = 0.04) and from an ancestor of Sheko into Begait (with Gir, f3 = −0.001, *p* = 1.40 × 10^−10^) were confirmed of Wannan to Xuanhan and of Sheko to Begait.

**Figure 2:**
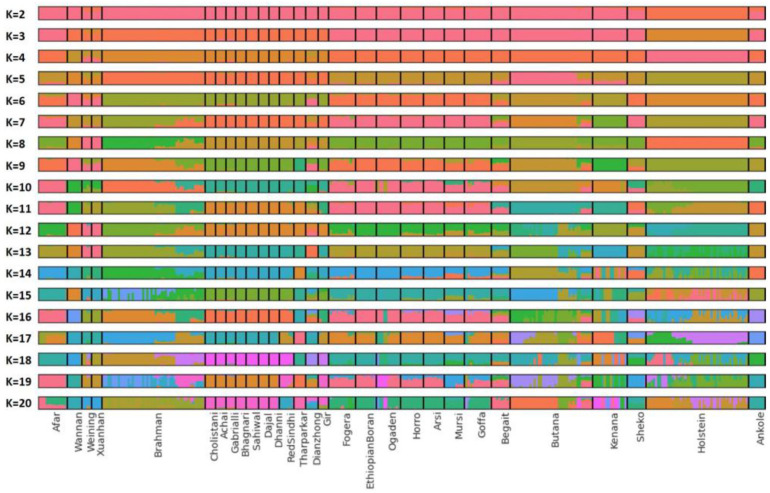
Admixture analysis of *Bos indicus* cattle breeds. Lowest cross validation error was estimated at K = 17. Seven Butana individuals showed admixture with other closely related breeds, whereas the remaining 33 Butana individuals showed no clear signs of admixture.

Out of the 40 investigated Butana cattle, seven individuals clustered with Kenana and Begait cattle (Figure 1b). These same seven individuals also exhibited signs of admixture with Kenana and other closely related breeds such as Begait (Figure 2). In contrast, the remaining 33 Butana cattle formed a distinct cluster in the PCA and did not show clear evidence of admixture based at K = 17, although three distinct subgroups emerged in different colors, which may suggest a relatively pure Butana group. These individuals originated from various locations, indicating that the observed genetic patterns are more plausibly attributed to historical gene flow rather than recent relatedness, shared environment, or inbreeding. Therefore, subsequent analyses focused on the 33 individuals displaying minimal signs of admixture.

### 3.2. High Diversity and Low Inbreeding Detected in Butana Cattle

**Figure 3:**
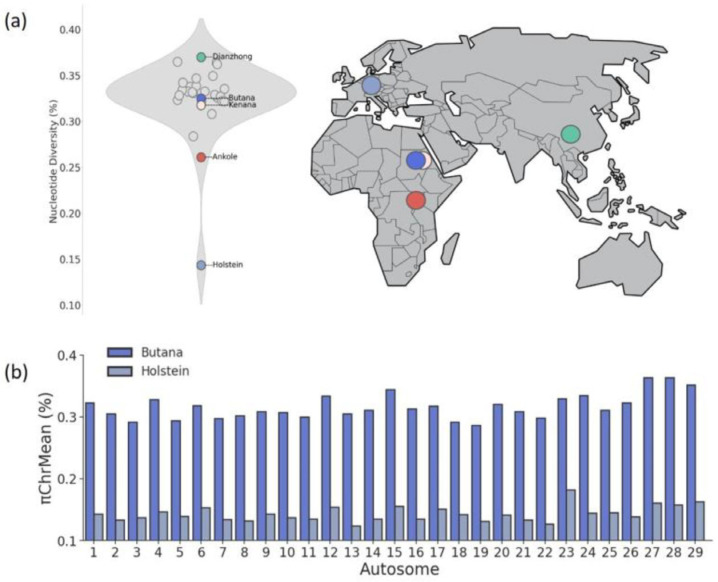
Diversity among *Bos indicus* cattle breeds with Holstein as outgroup. (**a**) Total mean nucleotide diversity across all investigated cattle breeds. Colors indicate Butana (blue), Kenana (rose), the breed with the highest (green) and lowest (red) total nucleotide diversity, and Holstein (violet) as outgroup. (**b**) Mean nucleotide diversity per chromosome in Butana (blue) and Holstein (violet). While Holstein samples in this study include individuals from both Europe and North America, the map highlights their ancestral region of origin.

Total mean nucleotide diversity π_TotMean_ ranged from 0.26 ± 0.01% in Ankole (or 0.14 ± 0.01% in Holstein which served as the outgroup) to 0.37 ± 0.02% in Dianzhong (Figure 3a, Table S1). Butana cattle (π_TotMean_ = 0.32 ± 0.02%) exhibited a level of genetic diversity comparable to that of most indicine breeds (mean π_TotMean_ = 0.33 ± 0.02% across breeds). The mean π_TotMean_ of indicine breeds was 2.4 times higher nucleotide diversity compared to Holstein. Interestingly, Butana and most other *B. indicus* breeds showed the highest nucleotide diversity per chromosome (π_ChrMean_) on chromosomes 27, 28, and 29 (Figure 3b), whereas Holstein showed the highest π_ChrMean_ on chromosome 23. Furthermore, the differences in π_ChrMean_ between chromosomes was less pronounced in indicine breeds compared to Holstein.

**Figure 4:**
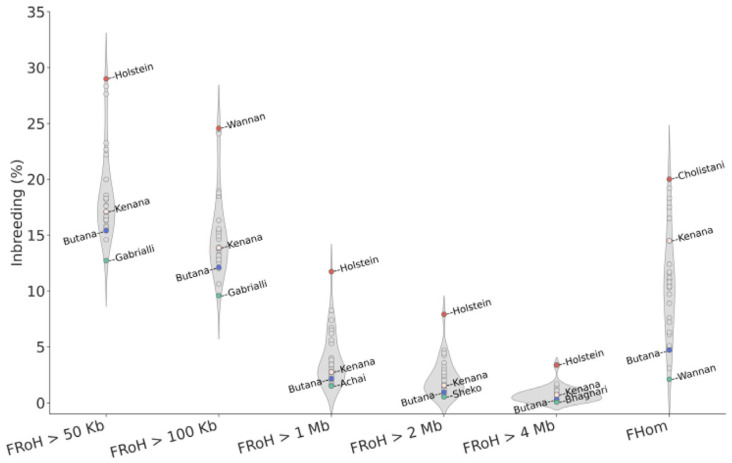
Genomic inbreeding among *Bos indicus* cattle breeds. Inbreeding estimated from Runs of Homozygosity (RoH) considering RoH lengths from >50 Kb to >4 Mb, and excess of homozygosity (F_Hom_). Inbreeding values for Butana and Kenana are highlighted in blue and rose, respectively, the highest inbreeding values are highlighted in red and lowest in green.

With regard to inbreeding, F_Hom_ ranged from 2.1 ± 1.7% in Chinese Wannan to 20.0 ± 19.2% in Cholistani (a breed from Pakistan) and Butana was observed with a F_Hom_ of 4.7 ± 3.0% (Figure 4, Table S1). Ancient inbreeding in terms of F_RoH > 50 kb_ ranged from 12.7 ± 7.3% in Gabrialli (a breed from Pakistan) to 29.0 ± 3.98% in Holstein, while more recent inbreeding events in terms of F_RoH > 4 Mb_ ranged from 0.06 ± 0.09% in Bhagnari (a breed from Baluchistan Province) to 3.4 ± 2.3% in Holstein. In Butana, low F_RoH_ values were observed across all RoHs lengths categories (F_RoH > 50 Kb_ = 15.4 ± 2.2%, F_RoH > 100 Kb_ = 12.1 ± 2.3%, F_RoH > 1 Mb_ = 2.2 ± 1.8%, F_RoH > 2 Mb_ = 1.0 ± 1.2%, and F_RoH > 4 Mb_ = 0.3 ± 0.6%), providing evidence for low recent and ancient inbreeding events.

### 3.3. Butana’s Unique Variants May Hold Key to Heat Stress Adaptation and Immune Function

In total, 93,899 variants unique to Butana cattle were detected. These variants were polymorphic in pure Butana cattle but monomorphic in all other 28 investigated breeds. Out of those, only 553 variants had a MAF > 0.05 and were predicted *in silico* to have a moderate or high impact on gene transcripts (Table S3). Most noteworthy were variants predicted to have high impact on gene transcripts as they are assumed to significantly affect the function of the gene product. These include 3 start lost, 25 stop gained, and 80 frameshift mutations. Four of these high impact variants are particularly interesting as they are quite frequent in the Butana cattle. The Butana-specific novel variant on chromosome 1 at 151,888,412 bp had a MAF of 0.11 and results in a stop gained mutation in *COL6A5* (collagen type VI alpha 5 chain) gene. The others were three frameshift variants: one on chromosome 23 at 27,525,516 bp with a MAF of 0.09 affecting the gene *HSPA1L* (heat shock 70kDa protein 1-like), which is part of cellular response to heat stress pathway (R-BTA-2262752, Reactome), and two insertions on chromosome 5 at 109,393,293 bp and 109,393,261 bp had both a MAF of 0.09 and are affecting the gene *TUBA8* (tubulin alpha 8). Furthermore, genes harboring variants that were unique to Butana, had a MAF ≥ 0.05, and showed moderate or high impact on gene transcripts, were checked for enrichment with GO terms as well as Reactome and KEGG pathways. Significantly enriched pathways were found in Reactome including immune system (R-BTA-168256, *p*_adj_ = 2.16 × 10^−3^), and homeostasis (R-BTA-109582, *p*_adj_ = 4.29 × 10^−2^), and in KEGG including ECM-receptor interaction (04512, *p*_adj_ = 8.94 × 10^−3^), focal adhesion (04510, *p*_adj_ = 1.92 × 10^−2^), and toxoplasmosis (05145, *p*_adj_ = 3.07 × 10^−2^) (Table S4).

### 3.4. Signatures of Selection Indicate Potential Adaptation to Immune Response in Butana

**Figure 5:**
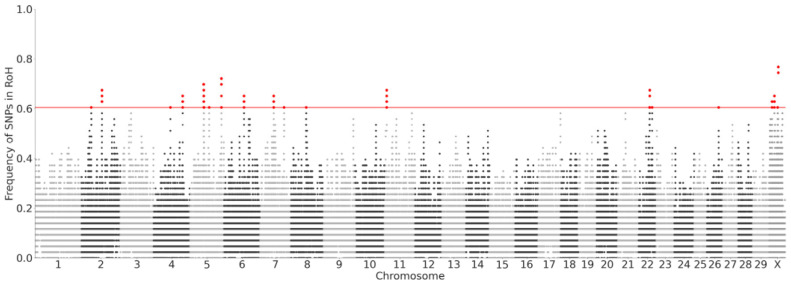
Runs of Homozygosity in pure Butana. The variants above the top 0.05 percentile of RoH frequencies are highlighted in red.

Regions with a high density of RoHs, referred to as RoH islands, were detected on ten chromosomes in pure Butana cattle (Figure 5, Table S5). Genes from three of these RoH islands were significantly enriched for GO terms. The RoH island on chromosome 5 harbors the genes *IRAK3* (Interleukin 1 receptor associated kinase 3), *CHADL* (Chondroadherin like), and *POLR3B* (RNA polymerase III subunit B). On chromosome 11, the RoH island includes the gene *IL18RAP* (Interleukin 18 receptor accessory protein), while the RoH island on chromosome 26 contains *RAB11FIP2* (RAB11 family interacting protein 2). These genes were significantly enriched (p_adj_ < 0.05) in a range of GO terms associated with immune response pathways (Table S6), including positive regulation of innate immune response (GO:45089), positive regulation of response to biotic stimulus (GO:02833), regulation of innate immune response (GO:45088), positive regulation of defense response (GO:31349), positive regulation of response to external stimulus (GO:32103), toll-like receptor signaling pathway (GO:02224), and regulation of response to biotic stimulus (GO:02831).

**Figure 6:**
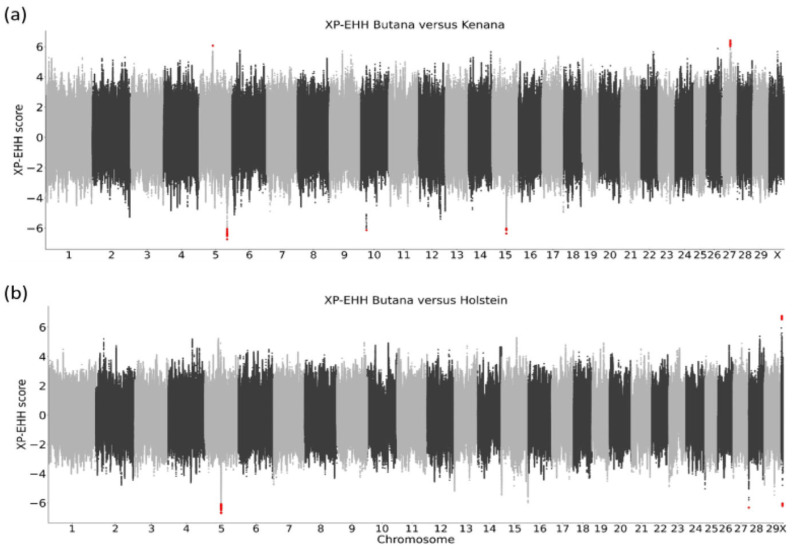
Signatures of selection in Butana compared to Kenana or Holstein cattle. (**a**) Butana versus Kenana. (**b**) Butana versus Holstein. Significant variants (*p*-values < 0.05 after Bonferroni correction) are highlighted in red indicating genomic regions under putative selection.

When comparing signatures of selection between Butana and Kenana, which is the closest related breed to Butana, five genomic regions showed significance (Figure 6a, Table 1). In Butana, two regions showing signatures of selection were detected on chromosomes 5 and 27. On chromosome 5, the region contained one variant downstream of *XPOT* (exportin for tRNA) and four additional flanking genes *TBK1* (TANK binding kinase 1), *C5H12orf56* (C12orf56 homolog), *KICS2* (KICSTOR subunit 2), and *SRGAP1* (SLIT-ROBO rho GTPase activating protein 1). On chromosome 27, ten variants formed the selection island surrounding *PPP1R3B* (protein phosphatase 1 regulatory subunit 3B) and *TNKS* (tankyrase).

In Kenana cattle compared to Butana, three regions showing signatures of selection were identified on chromosomes 5, 10, and 15. The region on chromosome 5 harbors nine genes including *CD163L1* (CD163-like 1), *PEX5* (peroxisomal biogenesis factor 5), *RBP5* (retinol binding protein 5), and *C1RL* (complement C1r subcomponent-like). The region on chromosome 10 harbors *TRAV24* (T cell receptor alpha variable 24) and five additional uncharacterized genes, while the region on chromosome 15 contains 15 olfactory receptor genes and five uncharacterized genes.

When comparing signatures of selection between Butana and Holstein cattle (Figure 6b, Table 1), only one region was detected in Butana on chromosome X. This region of selection contained 12 sequence variants and contained 13 genes including *FAM50A* (family with sequence similarity 50 member A), *PLXNA3* (plexin A3), *LAGE3* (L antigen family member 3), *SLC10A3* (solute carrier family 10 member 3), *FAM3A* (family with sequence similarity 3 member A), *G6PD* (glucose-6-phosphate dehydrogenase), *IKBKG* (inhibitor of nuclear factor kappa B kinase regulatory subunit gamma), and seven uncharacterized genes. In Holstein cattle, compared to Butana, three regions showing selection signatures were identified (Figure 6b, Table 1). On chromosome 5, a selection region containing 15 variants was flanked by nine olfactory receptor genes belonging to the olfactory receptor family 9, as well as *NEUROD4* (neuronal differentiation 4) and two uncharacterized genes. On chromosome 28, a region defined by a single variant showed a signature of selection. This variant was located near four genes, including three olfactory genes belonging to the olfactory receptor family 5, and one uncharacterized gene. On the X chromosome, five variants formed a region of selection flanked by *YIPF6* (Yip1 domain family member 6), *OPHN1* (oligophrenin 1), and one uncharacterized gene.

### 3.5. Pathogen Responsible for Tropical Theileriosis Was Detected in Butana Cattle

Sequencing reads from the 22 sequenced Butana that did not map to the *B. taurus,* the current reference genome available for cattle, were screened for taxonomy assignment to other species. In total, 6765 different species were detected in at least one of 22 sequenced Butana. Among these, the most abundant and prevalent were different *Bosea* species, a common gram-negative bacteria genus present in the environment (Table 2). Interestingly, a protozoa known to cause economic losses and impact cattle production in Northern Africa, *Theileria annulata*, was detected in all Butana cattle. In addition, another pathogen that can cause diseases in cattle was *Burkholderia cenocepacia*. *B. cenocepacia* can infect cattle, leading to various health issues, including mastitis and weak calves syndrome [41,42]. This bacteria was also observed in all sequenced Butana cattle, with an average of 14,410 reads per animal.

## 4. Discussion

This study provides a comprehensive genomic overview of Butana cattle, offering new insights into their genetic diversity and inbreeding, relationship with other *B. indicus* breeds, and adaptive traits. We identified selection signatures, candidate genes, and unique variants that reflect the distinct evolutionary path of Butana cattle. While some of the immune-related selection signals have been reported in Butana and Kenana populations previously, our study extends these findings by applying whole-genome sequencing data and by analyzing Butana as a distinct breed rather than in combination with other Sudanese populations. This higher-resolution approach reveals additional diversity and provides a more detailed view of the genetic architecture underlying adaptation. These findings contribute to our understanding of how environmental pressures, management practices, and historical factors have shaped the genome of this important indigenous breed.

### 4.1. Genetic Affinity and Historical Introgression

The observed genetic relationships between Butana cattle and the other investigated African *B. indicus* breeds, particularly those from Sudan and Ethiopia, reflect a shared ancestry and historical connectivity within the region. The close genetic proximity to breeds such as Kenana, Begait, and Fogera, was supported by maximum likelihood trees, PCA, and F_ST_ statistics. The admixture signal of a subset of Butana individuals clustering with Kenana and Begait likely indicates historical gene flow. As these individuals originated from different locations, recent kinship or localized population structure is unlikely, supporting the hypothesis of older introgression events. These findings are consistent with previous studies highlighting Sudan and Ethiopia as historical corridors for zebu gene flow and cattle migration [4,43,44].

### 4.2. Diversity and Inbreeding

Butana cattle exhibit high genetic diversity, similar to other zebu cattle, and substantially greater than that observed in Holstein and other taurine breeds [45]. This pattern is consistent with the known effects of intensive selection and closed breeding practices in commercial taurine populations. The high genetic diversity in Butana is also mirrored by low levels of both recent and ancient inbreeding as indicated by homozygosity and RoH analyses. These findings highlight the genetic value of indigenous breeds like Butana, which may carry alleles important for resilience, disease resistance, and adaptation to harsh environments. Interestingly, the distribution of nucleotide diversity across chromosomes revealed distinct patterns between indicine and taurine breeds. While the nucleotide diversity was extremely high and almost uniformly distributed across all chromosomes in indicine breeds, the highest diversity in taurine breeds was observed on chromosome 23, a chromosome that harbors immune-related genes as part of the *BoLA* (bovine leukocyte antigen) complex [46]. Nucleotide diversity on chromosome 23 in Holstein was about 50% lower than that observed in Butana. The elevated diversity in Butana may reflect a larger historical effective population size, differences in recombination rates, lineage-specific demographic histories, and selection pressures, particularly on genes related to immune function. Although the sample size was moderate, the inclusion of animals from eight geographically distinct locations and additional genomes from public databases provides a representative snapshot of the genetic diversity within the Butana breed.

### 4.3. Candidate Genes and Pathways for Heat Adaptation in Butana Cattle

The high number of unique variants in Butana cattle, including those with predicted functional relevance, highlights their distinct genomic profile and potential adaptation to hot climates. Among the affected genes, four genes *COL6A5*, *HSPA1L*, *TUBA8*, and *XPOT* emerged as promising candidates genes that may directly or indirectly contribute to the heat adaptation. *COL6A5*’s role in extracellular matrix stability might contribute to maintaining tissue integrity and elasticity under thermal stress, facilitating proper tissue function despite environmental challenges. This gene has been previously reported to affect cashmere formation and development in antelope, ibex, and goats [47] and wool in sheep [48]. This gene has also been reported to be upregulated in skin development in Dezhou donkeys [49]. Furthermore, variations in *COL6A5* were linked to dermal phenotypes in humans and skin development in other species [50,51,52]. Although direct evidence in cattle is limited, *COL6A5* might be involved in coat characteristics, which play a crucial role in thermoregulation and protection from solar radiation. *HSPA1L*, a heat shock protein, is directly involved in protecting cellular proteins from heat-induced denaturation and facilitating cellular stress response mechanisms. A novel, Butana-specific frameshift variant in *HSPA1L* affecting its only exon may alter gene function, although the exact impact remains to be clarified. Prior studies have linked indels in *HSPA1L* to fertility [53,54] and cell survival under heat stress [55,56], highlighting its relevance for thermal resilience. *TUBA8*, a gene involved in cytoskeletal stability and muscle development, has been associated with growth and skeletal adaptation in pigs [57] and yaks [58], which are living in extreme conditions in the Qinghai-Tibet Plateau. Frameshift variants in *TUBA8* could reflect adaptations maintaining cellular integrity under heat stress. Both *TUBA8* and *XPOT*, involved in fundamental cellular processes such as protein transport and cytoskeletal stability, could support maintaining cellular integrity and function during heat stress, preventing damage that might otherwise compromise activity and productivity. Notably, *TUBA8* expression has been previously reported to be enriched and increase in bovine oocytes during seasonal heat stress [59], reinforcing its potential role in thermal resilience. The presence of two frameshift variants in *TUBA8* exons with moderate allele frequency in Butana further supports the hypothesis of selection-driven adaptation to hot environments through enhanced cellular robustness.

### 4.4. Immune-Related Selection Signatures in Butana Cattle

Pathway enrichment analyses of Butana-unique variants and functional enrichment analyses along with signatures of selection, consistently pointed toward immune-related processes as key targets of selection in Butana cattle. Significant enrichment was observed in pathways linked to immune response, homeostasis, cellular adhesion, and environmental adaptation, suggesting that Butana may harbor unique alleles contributing to thermal resilience (see above) and disease resistance. Several identified candidate genes such as *IRAK3*, *IL18RAP*, *CHADL*, *POLR3B*, and *RAB11FIP2* are directly involved in immune signaling, inflammatory responses, and cellular defense mechanisms in humans [60,61,62,63,64]. These genes, together with the overrepresentation of immune-related GO terms, indicate that immune-relevant genes in Butana cattle may have been shaped by natural or historical selection, potentially in response to regional pathogen pressure (e.g., such as bovine trypanosomiasis [65]) and/or harsh environmental conditions. This finding supports previous research where immune pathways were identified as signatures of positive selection for Butana and Kenana cattle [14].

### 4.5. Signatures of Selection Between Butana and Kenana Cattle

Several genomic regions showed signatures of selection in Butana when compared to the closely related Kenana breed, despite their shared ancestry. Notably, these regions included the genes *XPOT*, *TBK1*, *SRGAP1*, *PPP1R3B*, and *TNKS*. *XPOT* was previously identified within RoH islands of *B. indicus* cattle adapted to hot and semi-arid environments [66], suggesting a potential role in cellular stress responses under thermal conditions. *TBK1* seems to play a role in the induction of IFN-β and microbial infections [67,68], while *SRGAP1* has been linked to meat quality and feed efficiency traits in Nelore cattle [69], indicating potential dual relevance for both adaptation and productivity.

In another region, selection signals flanked *PPP1R3B* and *TNKS*, two genes previously associated with milk fat percentage in taurine breeds such as Holstein [70] and German Black Pied cattle [71]. Given Butana’s reputation for producing milk with high fat content, these loci may contribute to this economically important trait in an indicine context. In Kenana cattle, several genes under selection are associated with immune function, including *CD163L1*, *PEX5*, *RBP5*, *C1RL* [72,73,74,75], and *TRAV24*, which plays a key role in T-cell-mediated antigen recognition [76]. These findings suggest adaptation to breed-specific immune challenges. Additionally, a cluster of olfactory receptor genes under selection may reflect ongoing sensory adaptation, though such regions are common and should be interpreted with caution [77]. Although Butana and Kenana cattle are genetically similar, they are raised under contrasting environmental conditions within Sudan: Butana in hotter, more arid regions and Kenana in comparatively milder climates. The selection signals in Butana likely reflect adaptation to heat and harsher conditions, supporting the hypothesis that environmental pressures have shaped specific genomic regions related to both heat resilience and production traits.

### 4.6. Signatures of Selection Between Butana and Holstein Cattle

When comparing selection signals between Butana and Holstein cattle, distinct patterns emerged that reflect their contrasting selection histories and environmental conditions. In Butana, selected genes were primarily associated with metabolic regulation, stress response, and immune function. For instance, *G6PD* plays a central role in redox balance and cellular protection against oxidative stress and *G6PD* deficiency can cause acute hemolytic anemia in humans [78]. Further, *FAM3A* contributes to insulin signaling and energy metabolism [79,80], while *SLC10A3* is involved in bile acid transport, potentially affecting lipid digestion and metabolic efficiency [81]. The immune-related gene *IKBKG*, a key regulator of the NF-κB pathway, is essential for inflammation control and cellular stress response [82,83]. Other genes, such as *FAM50A* [84,85,86] and *PLXNA3* [87], are linked to transcriptional regulation and neural development, which may further support physiological resilience in challenging environments. In contrast, selection signals in Holstein cattle included genes associated with sensory perception and neural development. The presence of multiple olfactory receptor genes suggest ongoing adaptation in sensory functions, although such gene families are known to evolve rapidly across mammals and may also reflect genomic instability or drift [77,88,89]. Additional selected genes such as *YIPF6* and *OPHN1* point to intracellular transport and neuronal processes. *YIPF6* supports proper protein trafficking within the endomembrane system and may influence immune signaling and cell maintenance [90,91]. *OPHN1*, involved in cytoskeletal regulation and brain development, has been associated with behavioral and neurological traits [92], which could reflect selection for temperament or stress response under intensive production systems. Taken together, these contrasting patterns suggest that while Holstein cattle have been shaped predominantly by artificial selection for productivity and possibly behavior, Butana cattle exhibit genomic signatures indicative of natural selection for heat tolerance, metabolic efficiency, and immune robustness, traits critical for survival and performance in arid environments. Our results of genomic regions of selection signatures and Butana specific genetic variation repeatedly hint on immune response genes and the pathways they act in. These findings are consistent with recent studies in other cattle breeds and mammals, which provide evidence for the critical role of the bodily immune response to stress that forms the backbone of resilience against environmental stress challenges. A large-scale genomic study in Angus and Hanwoo cattle recently has identified numerous selection signatures, including many genes associated with immunity and adaptation to environmental stress [93]. Similarly, comprehensive studies in human populations including Neanderthals demonstrate that the human immune response has been shaped by natural selection and adaptation to environmental stressors, highlighting the central role of the immune system in environmental adaptation and resilience [94].

### 4.7. Endemic Pathogens Detected in Butana Cattle

The detection of *T. annulata* in all sequenced Butana cattle, with consistently high read counts, indicates the presence of tropical theileriosis within the Butana population. This tick-borne parasite causes clinical symptoms such as fever, anemia, and reduced milk yield, and has been previously reported as endemic in Sudanese cattle, including Butana [95,96,97,98]. Its widespread presence underlines the need for improved management and control strategies to protect animal health and productivity. Similarly, the identification of *B. cenocepacia* across all animals, although at lower abundance, is notable. This opportunistic pathogen is known to cause mastitis and the birth of weak calves syndrome [41,42,99], potentially negatively affecting animal welfare and reproductive efficiency in Butana herds. Importantly, these observations were derived from the analysis of unmapped sequencing reads and should, therefore, be interpreted as pathogen DNA signatures rather than confirmed infections. Such signals may reflect environmental exposure or background microbial DNA rather than active disease. Validation through serological or veterinary analyses are necessary to confirm infection status. Nonetheless, this approach highlights the hidden potential of metagenomic data to reveal biologically relevant and often overlooked information about livestock health. Integrating such metagenomic screening into livestock genomics could thus provide valuable insights into pathogen dynamics and support future genomic surveillance and herd health monitoring efforts.

### 4.8. Breeding and Conservation Implications

The results presented here have direct relevance for breeding and conservation strategies. The high genetic diversity and low inbreeding levels observed in Butana cattle underline their value as a genetic resource for resilience traits, particularly heat tolerance and disease resistance. These adaptive characteristics could be strategically incorporated into breeding programs, for instance through genomic selection or crossbreeding approaches, to improve productivity under climate and pathogenic stress. Furthermore, conserving the genetic integrity of Butana cattle is essential to preserve alleles contributing to environmental adaptability, which may become increasingly important in the context of global warming and changing production environments.

## 5. Conclusions

This study provides a comprehensive genomic characterization of Butana cattle, revealing high genetic diversity, low levels of inbreeding, and distinct signatures of selection potentially associated with heat tolerance, metabolic adaptation, and immune function. These findings underscore the adaptive value of the Butana genome and its potential relevance for breeding programs focused on enhancing resilience to climate extremes and disease pressure. In particular, the discovery of unique genetic variants and selection signals in genes related to heat stress and immune defense highlight Butana’s potential as a genetic resource for improving livestock performance in challenging environments. Furthermore, the identified immune-related loci may be linked to resistance to diseases such as bovine trypanosomiasis, and their broader contribution in resilience to environmental challenges warrants further investigation into their functional consequences.

## Figures and Tables

**Table 1:** Regions of selection signatures in Butana, Kenana, and Holstein cattle. For each region (chromosome: start–end), the length in kilobases (kb), the number of variants, and the flanking genes within ±250 kb are listed.

Region of Signature Selection	Length (kb)	No. of Variants	Flanking Genes ±250 kb
Butana (versus Kenana)			
5: 49,459,819–49,459,819	0.00	1	*TBK1*, *XPOT*, *C5H12orf56*, *KICS2*, *SRGAP1*
27: 25,367,215–25,367,462	0.25	10	*PPP1R3B*, *TNKS*
Kenana (versus Butana)			
5: 103,099,474–103,100,164	0.69	13	ENSBTAG00000039256, ENSBTAG00000050301, *CD163L1*, *PEX5*, *CLSTN3*, *RBP5*, *C1RL*, ENSBTAG00000050749, ENSBTAG00000037743
10: 23,772,862–23,772,862	0.00	1	ENSBTAG00000051554, ENSBTAG00000048374, ENSBTAG00000052580, ENSBTAG00000048874, *TRAV24*, ENSBTAG00000052314
15: 46,039,792–46,085,328	45.54	4	*OR2D2*, *OR10A4*, *OR10A5*, *OR10A5L*, *OR10A5G*, *OR6A2*, *OR6B18*, ENSBTAG00000027525, *OR6B17*, ENSBTAG00000037603, *OR2D4*, *OR2D3G*, *OR2AG1E*, *OR2AG1G*, *OR2AG1*, *OR2AG2*, *OR2D37*, ENSBTAG00000051394, ENSBTAG00000037937, ENSBTAG00000049294
Butana (versus Holstein)			
X: 37,758,460–37,758,587	0.13	12	*FAM50A*, *PLXNA3*, *LAGE3*, ENSBTAG00000014331, *SLC10A3*, *FAM3A*, *G6PD*, ENSBTAG00000053534, *IKBKG*, ENSBTAG00000001900, ENSBTAG00000048914, ENSBTAG00000055292, ENSBTAG00000053848, ENSBTAG00000052652
Holstein (versus Butana)			
5: 59,654,384–59,657,407	3.02	15	*OR9K2I*, *OR9K2H*, *OR9K2K*, *OR9K2C*, ENSBTAG00000045722, ENSBTAG00000054855, *OR9K2*, *OR9K15*, *OR9K1*, *OR9K2F*, *OR9K1B*, *NEUROD4*
28: 319,705–319,705	0.00	1	ENSBTAG00000038418, *OR5D18K*, *OR5L20*, *OR5AS1*
X: 82,098,289–82,098,601	0.31	5	*YIPF6*, *OPHN1*, ENSBTAG00000052786

**Table 2:** Microorganisms detected in Butana cattle with mean number of >5000 reads. Min and Max refer to minimum and maximum number of reads detected in one animal classified for a certain taxon. Summary statistics includes taxon name, NCBI taxon ID, minimum and maximum read counts, number of samples (n), mean read count, and standard deviation read count (SD).

Taxon Name	Taxon ID	Min	Max	n	Mean	SD
*Bosea vestrisii*	151416	17	196,724	12	131,272	79,955
*Bosea* sp. *Tri-49*	1867715	14	236,073	15	125,753	106,967
*Ralstonia mannitolilytica*	105219	5419	278,625	22	78,512	103,706
*Theileria annulata*	5874	7282	259,012	22	71,638	72,619
*Botrytis cinerea*	40559	10,118	173,623	8	54,423	67,049
*Bosea beijingensis*	3068632	35	23,161	10	18,501	6651
*Bosea* sp. *(in: a-proteobacteria)*	1871050	142	20,174	10	16,335	5824
*Bosea* sp. *F3-2*	2599640	33	22,410	11	16,332	8192
*Variovorax paradoxus*	34073	70	22,751	22	14,694	6100
*Burkholderia cenocepacia*	95486	54	169,760	22	14,410	39,918
*Novosphingobium* sp. *EMRT-2*	2571749	6096	19,721	21	14,149	3119
*Bosea* sp. *NBC_00550*	2969621	20	18,145	11	13,299	6647
*Bosea* sp. *UC22_33*	3350165	54	16,451	10	13,079	4723
*Bosea* sp. *RAC05*	1842539	30	15,242	10	12,217	4404
*Bosea* sp. *PAMC 26642*	1792307	26	14,504	10	11,357	4123
*Bosea* sp. *ANAM02*	2020412	26	15,277	11	11,247	5627
*Stenotrophomonas maltophilia*	40324	121	36,873	22	10,556	8575
*Bosea* sp. *685*	3080057	41	12,778	10	10,175	3667
*Bosea vaviloviae*	1526658	39	12,369	10	9623	3473
*Bosea* sp. *AS-1*	2015316	20	13,504	12	9115	5528
*Microviridae* sp.	2202644	12	24,457	3	8162	14,112
*Variovorax* sp. *UC74_104*	3374555	49	13,082	22	7929	3911
*Variovorax* sp. *V15*	3065952	46	9775	22	5910	2887

## Data Availability

The raw sequencing fastq files generated in the current study were made publicly available in the European Nucleotide Archive (PRJEB94940).

## Supplementary Materials

The following supporting information can be downloaded at: https://www.mdpi.com/article/10.3390/genes16121429/s1; Table S1: Summary statistics of genetic diversity and runs of homozygosity (RoH) in the investigated cattle breeds; Table S2: Results of outgroup f3-statistics testing shared genetic drift between pairs of cattle populations; Table S3: Summary of 553 variants unique to Butana cattle with MAF > 0.05 and moderate or high impact on gene transcripts; Table S4: KEGG and Reactome terms enriched among genes harboring Butana-specific variants with minor allele frequency (MAF) > 0.05 and predicted moderate or high impact on transcripts; Table S5: Genomic regions with a high density of runs of homozygosity (RoHs), referred to as RoH islands, identified in pure Butana cattle; Table S6: Gene ontology (GO) molecular function terms enriched among genes in RoH islands of Butana cattle.
